# Distribution of body mass index in children with different parental risk: Findings of a family-based cohort study in a West-Asian population

**DOI:** 10.1038/s41598-019-45543-y

**Published:** 2019-06-28

**Authors:** Leila Cheraghi, Parisa Amiri, Mehrdad Karimi, Yadollah Mehrabi, Fereidoun Azizi

**Affiliations:** 1 Department of Epidemiology and Biostatistics, Research Institute for Endocrine Sciences, Shahid Beheshti University of Medical Sciences, Tehran, Iran; 2 Research Center for Social Determinants of Health, Research Institute for Endocrine Sciences, Shahid Beheshti University of Medical Sciences, Tehran, Iran; 3 Department of Epidemiology and Biostatistics, School of Public Health, Tehran University of Medical Sciences, Tehran, Iran; 4 Department of Epidemiology and Biostatistics, School of Public Health and Safety, Shahid Beheshti University of Medical Sciences, Tehran, Iran; 5 Endocrine Research Center, Research Institute for Endocrine Sciences, Shahid Beheshti University of Medical Sciences, Tehran, Iran

**Keywords:** Epidemiology, Paediatric research

## Abstract

Using quantile regression analysis, the current study, conducted within the framework of the Tehran Lipid and Glucose Study (TLGS) aimed to assess the effects of parental risk clusters on different percentiles of Body Mass Index (BMI) distribution in children. Participants included 2296 school-aged children who had participated in the baseline assessment of the TLGS and were followed for an approximate duration of fifteen years. Parental socio-demographic, behavioral and clinical characteristics were considered to determine risk clusters. Comparing of the high- to the low-risk parental clusters showed that after adjusting for age in boys, BMI was significantly higher at the 75^th^ (1.82, p = 03), 85^th^ (1.78, p = 0.007) and 95^th^ (1.66, p = 0.03) percentiles; and in girls it was significantly higher at the 25^th^ (1.45, p = 0.003), 50^th^ (1.05, p = 0.015), 95^th^ (2.31, p = 0.018) and 97^th^ (2.44, p = 0.006) percentiles in the high risk cluster. Our data indicate that during a long-term follow up, children with a high-risk family are more likely to have higher BMI, compared to their counterparts in low-risk families, a difference observed mainly at the upper percentiles of BMI distribution for both genders and at all ages, findings that should be considered for strategies aimed at preventing childhood obesity and its consequences.

## Introduction

The worldwide rising trend of childhood overweight and obesity has been well documented over the past three decades^1^. According to the CASPIAN study conducted on a large national representative sample of Iranian children and adolescents, the prevalence of overweight and obesity among children aged 6–18 years, has been reported to be 10.1 and 4.79% respectively^2^. With the steep escalation in urbanization, Iran is currently undergoing a sharp rising trend in lifestyle change and nutritional transition, which could predispose more children to overweight and obesity in the future^3^. This trend is even more alarming when we consider the complications of obesity in children such as type 2 diabetes, high blood pressure, high cholesterol and other cardiovascular diseases^4^. Hence, identifying the factors influencing overweight and obesity in Iranian children is of prime importance.

Childhood obesity is a multi-factorial condition influenced by unhealthy lifestyles, genetic predisposition and the socio-economic- and cultural status^5^. Children’s lifestyles particularly for younger groups, are directly or indirectly affected by family environments, parents’ role and behavioral modeling^6^. Parents’ age, knowledge and education, employment status, physical activity and smoking are reported to be among the most important familial contributors to children weight status^7–10^. Beyond these parental socio-behavioral determinants, children, whose parents were both obese, are more likely to gain excessive weight, especially if the mothers were obese^11^. Further evidence shows that, compared with children whose parents did not have metabolic syndrome, those who had at least one parent with this syndrome had significantly higher levels of general-, particularly central-obesity and insulin resistance^12^. In this regard, several studies have demonstrated the parental determinants of overweight/obesity among Iranian children and adolescents^13,14^. Based on the results of a nationwide study conducted on 7908 adolescents, aged 11–19 years, parental overweight and obesity, education as well as the father’s job were the main influential factors of parental determinants of overweight in Iranian adolescents^13^.

Despite these well documented associations between parental factors and their children’s overweight, data available are mainly the results that have emerged from interpretation of children’s Body Mass Index (BMI) status, mostly defined according to the 85^th^ or 95^th^ BMI percentiles of international or national reference charts^15^. This reduction of BMI to a binary variable (normal/overweight) can lead to loss of valuable information on the entire BMI distribution, particularly its upper part that could be the basis for the designing and implementation of obesity prevention programs in the early years of life. To combat these limitations, numerous studies^16–19^ have aimed at investigating the association between parental factors and different percentiles of BMI, particularly for upper percentiles, using the quantile regression model, which has been considered as an effective analytical method for modeling quantiles of BMI distribution directly^19^.

While the effects of parental characteristics on the risk of non-communicable diseases and other health related outcomes in children have previously been documented^20–23^, little is known about the cluster effect of parental variables on the percentiles of BMI distribution. The present study is among the first efforts, aimed at (1) detecting different parental clusters which could potentially influence their children’s weight status and (2) assessing the association between the identified parental clusters and the BMI distribution of children, using quantile regression analysis. Findings of the current study may help researchers and policymakers to design more effective interventions to tackle excessive weight gain in the early years of life.

## Results

### Participant characteristics

The baseline mean age for boys (49.5%) and girls was 13.29 ± 2.98 and 13.26 ± 3.07 years respectively. Mean ages of fathers and mothers were 46.5 ± 8.2 and 39.8 ± 7.1 respectively.

### Parental clusters

**Figure 1:**
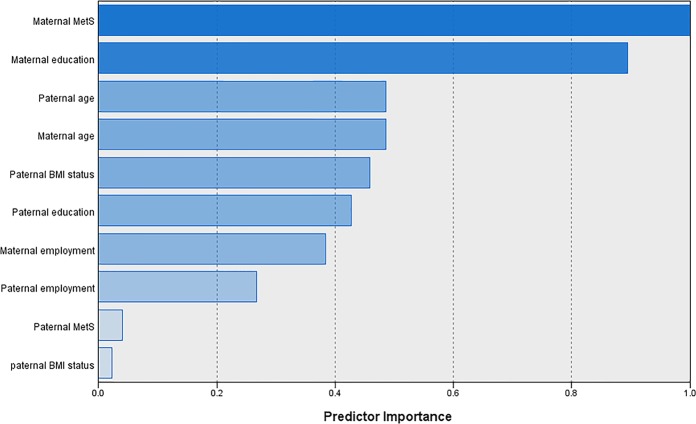
Importance of variables associated with parental classification.

As described in our previous study^23^, using cluster analysis and based on the distribution of the influential parental factors, including age, education, employment status, metabolic syndrome and body weight status, three distinct clusters were identified and labeled as the low-, moderate- and high-risk parental clusters. Considering the risk of overweight development, children in a specific cluster are similar in parental characteristics, whereas those in two different clusters differ in these characteristics. Parents’ smoking status and level of physical activity which were the least important factors were not included in the cluster analysis. The importance of the variables for the cluster solution was presented in Fig. 1 and results revealed that the most and the least important factors were maternal MetS and paternal obesity respectively.

A comparison of parental characteristics among the three identified clusters is shown in Table 1. Distribution of all variables included in the cluster analysis differed significantly among the three clusters (p < 0.001); the prevalence of maternal MetS was 0% in the low-risk parental cluster, compared to 11.0% in the high-risk parental cluster. All mothers in the low-risk parental cluster had secondary educational levels, whereas approximately half of them in the moderate- and high-risk parental clusters were either illiterate or had primary education. The low-risk parental cluster had the lowest means for paternal and maternal age, compared to the moderate- and high-risk parental clusters. Overall prevalence of maternal overweight and obesity was significantly higher in the high-risk parental cluster (93.2%), compared to the low- (61.4%) and moderate-risk (67.8%) ones (p < 0.001).

**Table 1:** Baseline characteristics of parents in the low, moderate and high risk parental clusters of overweight/obesity.

	Low-risk (N = 646)	Moderate-risk (N = 671)	High-risk (N = 979)	P-value
**Father’s characteristics**				
Age (years)	41.49 ± 5.10	45.86 ± 7.55	50.22 ± 8.42	<0.001
**Level of education**				
Illiterate	0 (0)	21 (3.1)	67 (6.8)	<0.001
Primary school	0 (0)	303 (45.2)	301 (30.7)	
Secondary school	511 (79.1)	237 (35.3)	471 (48.1)	
Higher	135 (20.9)	110 (16.4)	140 (14.3)	
**Occupation**				
Employed	643 (99.5)	631 (94.0)	729 (74.5)	<0.001
Unemployed with income	0 (0)	23 (3.4)	227 (23.2)	
Unemployed	3 (0.5)	17 (2.5)	23 (2.3)	
**Body weight status**				
Normal	241 (37.3)	297 (44.3)	309 (31.6)	<0.001
Overweight	294 (45.5)	278 (41.4)	493 (50.4)	
Obese	111 (17.2)	96 (14.3)	177 (18.1)	
**Metabolic syndrome**				
No	388 (57.0)	389 (58.0)	427 (43.6)	<0.001
Yes	278 (43.0)	282 (42.0)	552 (56.4)	
**Mother’s characteristics**				
Age (years)	35.51 ± 4.55	39.46 ± 6.61	42.95 ± 7.29	<0.001
**Level of education**				
Illiterate	0 (0)	14 (2.1)	63 (6.4)	<0.001
Primary school	0 (0)	316 (47.1)	407 (41.6)	
Secondary school	646 (100)	219 (32.6)	508 (51.9)	
Higher	0 (0)	122 (18.2)	1 (1)	
**Occupation**				
Employed	0 (0)	186 (27.7)	22 (2.2)	<0.001
Unemployed with income	0 (0)	8 (1.2)	21 (2.1)	
Unemployed	646 (100)	477 (71.1)	936 (95.6)	
**Body weight status**				
Normal	249 (38.5)	216 (32.2)	67 (6.8)	<0.001
Overweight	309 (47.8)	350 (52.2)	368 (37.6)	
Obese	88 (13.6)	105 (15.6)	544 (55.6)	
**Metabolic syndrome**				
No	646 (100)	611 (91.1)	871 (89.0)	<0.001
Yes	0 (0)	60 (8.9)	108 (11.0)	

Values are expressed as mean ± sd or number (%). P-value was assessed using Chi-square tests for categorical variables and ANOVA test for continuous variables.

### Sex- and age-specific curves of BMI percentiles according parental clusters

**Figure 2:**
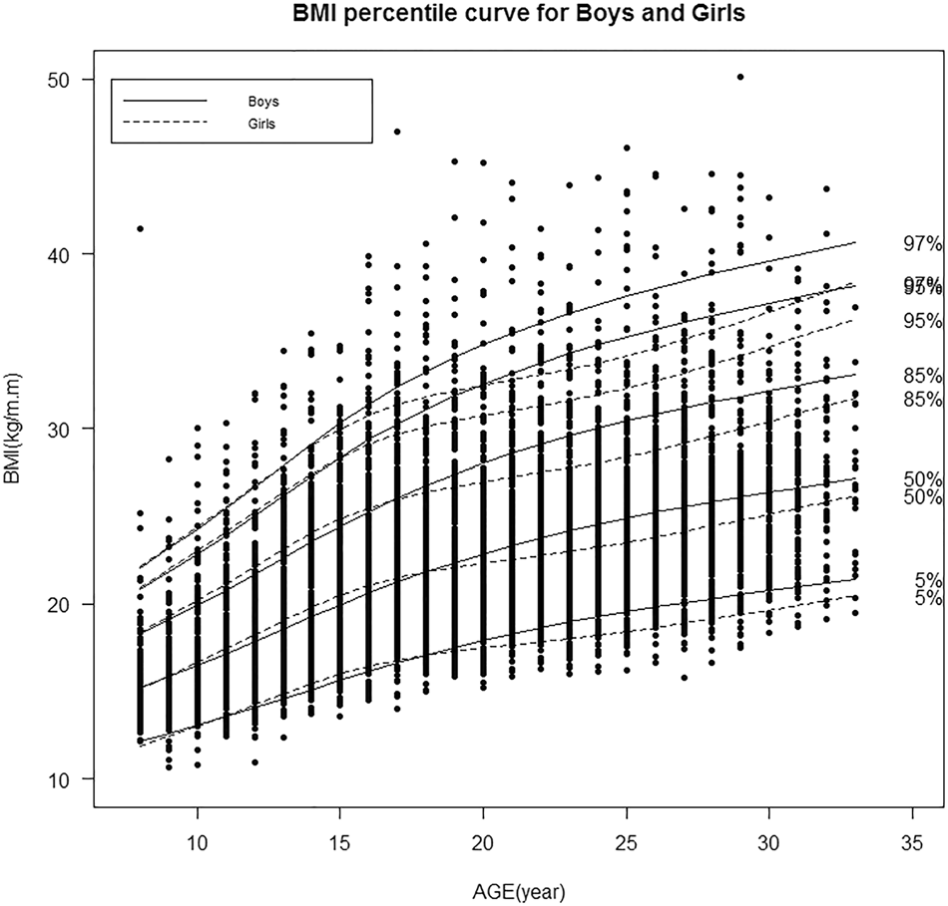
Gender- and age-specific BMI percentile curves during fifteen years of follow up.

**Figure 3:**
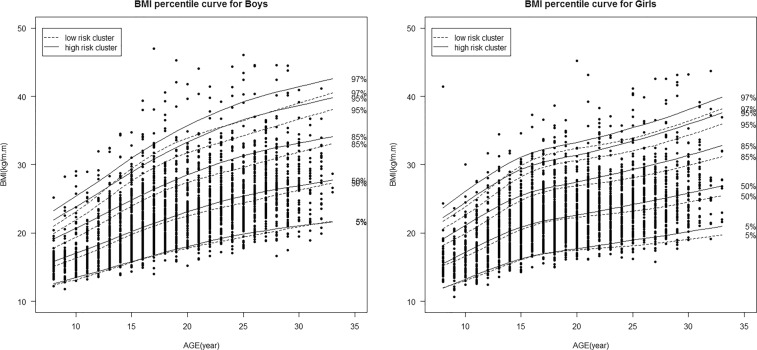
Gender-specific BMI percentile curves during fifteen years of follow up, according to children’s age and parental risk cluster.

The age-specific 5^th^, 50^th^, 85^th^, 95^th^ and 97^th^ percentile curves of BMI for all observations in boys and girls have been shown in Fig. 2. During the fifteen year follow-up, in both genders, all the percentile curves exhibited rising trends with increasing age; however, for boys these changes were more considerable in the higher percentile curves. Although curves of BMI percentiles had similar changes in both genders (approximately aged ≤ 15 years), it changed slightly for girls after sixteen years, compared to boys. The sex- and age-specific curves in the aforementioned BMI percentiles in the low- and high-risk parental clusters;the two groups which had the highest risk difference are presented in Fig. 3; results show that all of the percentile curves for the high-risk parental cluster are seen to be above the low-risk one. Differences observed between clusters were increased for the higher percentile levels in both genders and in all ages.

### Association between parental clusters and BMI distribution

Table 2 shows the results of the estimated coefficients and the 95% confidence intervals (CI) for the 5^th^, 25^th^, 50^th^, 75^th^, 85^th^, 95^th^ and 97^th^ percentiles of BMI in boys and girls. Results of quantile models show significant positive associations between all percentiles of BMI and age in both boys and girls (P < 0.001). Comparisons between the high- and low-risk parental clusters showed that, after adjusting for age, in boys BMI was significantly higher at the 75^th^ (1.82 kg/m^2^, 95% CI: 0.17–3.48, p = 0.03), 85^th^ (1.78 kg/m^2^, 95% CI: 0.49–3.08, p = 0.007) and 95^th^ (1.66 kg/m^2^, 95% CI: 0.20–3.13, p = 0.03) percentiles in the high-risk parental cluster. In girls there were significant differences between high- and low-risk parental clusters at the 25^th^ (1.45 kg/m^2^, 95% CI: 0.49–2.43, p = 0.003), 50^th^ (1.05 kg/m^2^, 95% CI: 0.21–1.88, p = 0.015), 95^th^ (2.31 kg/m^2^, 95% CI: 0.40–4.23, p = 0.018) and 97^th^ (2.44 kg/m^2^, 95% CI: 0.72–4.15, p = 0.006) percentiles of BMI. However, there was no significant difference between moderate- and low-risk parental clusters at any of the studied percentiles in either gender.

**Table 2:** Gender-specific quantile regression results for the different percentiles of BMI.

Coefficient^a^ (95% CI)^b^							
	5^th^	25^th^	50^th^	75^th^	85^th^	95^th^	97^th^
**Boys**							
**Model 1**							
Intercept	16.24 (15.54–16.94)	18.12 (17.07–19.17)	19.73 (19.37–20.09)	20.97 (20.21–21.74)	22.19 (21.41–22.98)	23.36 (22.19–24.19)	23.41 (22.55–24.27)
P values	<0.001	<0.001	<0.001	<0.001	<0.001	<0.001	<0.001
Age(years, centered at 13)	0.48 (0.45–0.52)	0.5 (0.48–0.53)	0.51 (0.49–0.53)	0.52 (0.49–0.55)	0.54 (0.51–0.57)	0.56 (0.51–0.61)	0.58 (0.51–0.66)
P values	<0.001	<0.001	<0.001	<0.001	<0.001	<0.001	<0.001
**Model 2**							
Intercept	16.16 (15.03–17.29)	16.78 (15.53–18.02)	19.92 (18.84–21.00)	20.74 (20.23–21.26)	21.49 (20.81–22.18)	22.85 (22.03–23.67)	22.90 (21.45–24.36)
P values	<0.001	<0.001	<0.001	<0.001	<0.001	<0.001	<0.001
Age(years, centered at 13)	0.50 (0.47–0.54)	0.51 (0.48–0.53)	0.52 (0.49–0.55)	0.53 (0.51–0.56)	0.55 (0.52–0.57)	0.51 (0.45–0.56)	0.54 (0.48–0.60)
P values	<0.001	<0.001	<0.001	<0.001	<0.001	<0.001	<0.001
Cluster (moderate vs. low)	−0.69 (−2.00–0.61)	1.30 (−0.55–3.15)	−0.68 (−1.85–0.50)	−0.29 (−0.91–0.31)	−0.67 (−2.18–0.83)	1.08 (−0.38–2.54)	1.07 (−0.95–3.09)
P values	0.29	0.17	0.26	0.34	0.37	0.15	0.29
Cluster (high vs. low)	0.70 (−0.67–2.07)	1.30 (−0.09–2.69)	0.04 (−1.58–1.67)	1.82 (0.17–3.48)	1.78 (0.49–3.08)	1.66 (0.20–3.13)	1.20 (−1.04–3.43)
P values	0.32	0.07	0.96	0.03	0.007	0.03	0.29
**Girls**							
**Model 1**							
Intercept	16.21 (15.72–16.69)	18.03 (17.19–18.87)	19.60 (19.16–20.03)	21.13 (20.76–21.51)	21.64 (21.11–22.17)	22.71 (21.45–23.98)	22.81 (20.55–25.07)
P values	<0.001	<0.001	<0.001	<0.001	<0.001	<0.001	<0.001
Age(years, centered at 13)	0.40 (0.36–0.44)	0.41 (0.38–0.44)	0.40 (0.38–0.43)	0.40 (0.37–0.43)	0.40 (0.37–0.43)	0.41 (0.35–0.46)	0.41(0.34–0.47)
P values	<0.001	<0.001	<0.001	<0.001	<0.001	<0.001	<0.001
**Model 2**							
Intercept	16.13 (15.33–16.91)	17.47 (16.58–18.36)	19.10 (18.29–19.70)	20.72 (19.59–21.84)	21.40 (20.28–22.53)	22.78 (21.54–24.03)	22.90 (21.64–24.16)
P values	<0.001	<0.001	<0.001	<0.001	<0.001	<0.001	<0.001
Age(years, centered at 13)	0.41 (0.37–0.45)	0.39 (0.37–0.41)	0.40 (0.37–0.42)	0.40 (0.37–0.43)	0.39 (0.36–0.43)	0.36 (0.31–0.41)	0.37 (0.31–0.43)
P values	<0.001	<0.001	<0.001	<0.001	<0.001	<0.001	<0.001
Cluster (moderate vs. low)	−0.22 (−1.20–0.75)	−0.35 (−1.39–0.68)	−0.44 (−1.73–0.85)	−0.16 (−1.75–1.43)	0.26 (−1.04–1.55)	−0.08 (−1.49–1.33)	0.05(−1.60–1.71)
P values	0.65	0.50	0.50	0.84	0.70	0.91	0.95
Cluster (high vs. low)	0.57 (−0.76–1.90)	1.45 (0.49–2.43)	1.05 (0.21–1.88)	0.64 (−0.73–2.02)	0.94 (−0.57–2.46)	2.31 (0.40–4.23)	2.44 (0.72–4.15)
P values	0.40	0.003	0.015	0.36	0.22	0.018	0.006

BMI = Body Mass Index, CI: confidence interval.

^a^The coefficient represents the difference in the value of BMI at the nth percentile for moderate- and high-risk parental clusters compare to low-risk. For age the coefficient represents the change in the value of BMI at the nth percentile for one year increase in age. The intercept is the value of the nth percentile of BMI when all variables are zero.

^b^Confidence intervals (CI) are based on 100 bootstrap samples.

P values were obtained using longitudinal quantile regression analysis.

## Discussion

This study was conducted to determine the sex- and age-specific BMI distribution of children aged 8–18 years, according to their parental risk clusters (high, moderate and low) during a 15 year follow-up of the TLGS. The current results identified three parental risk clusters i. e. the low-, moderate- and high-risk clusters based on socio-behavioral and cardio-metabolic factors and educational level as well as age in both parents. Our results show a sex-specific rising trend in all percentiles of BMI at all ages through the study duration. Using longitudinal quantile regression analysis, our data provide evidence that during the follow up, for both genders and at all ages the high-risk parental cluster was associated with higher BMI levels at all percentiles. While the significant difference between low- and high-risk parental clusters in boys was limited to the upper part of BMI distribution (75th, 85th and 95th percentiles), in girls the middle and lower percentiles (25th, 50th, 95th and 97th percentiles) were also included. There is no evidence regarding significant differences in BMI distribution between low- and moderate-risk parental clusters for both genders.

Based on the current results, in general, the upper percentiles of BMI were higher in boys than girls. A more detailed comparison between boys and girls showed an approximately similar rising trend in all age-specific percentiles of BMI for boys and girls aged ≤15 years. However, compared to boys, the BMI percentile curves for girls seemed to plateau untill almost 25 years, after which age, there was another increasing trend of BMI in both genders. The observed age- and gender-specific pattern of BMI percentile curves in our study is consistent with those of previous nationwide reports regarding BMI trends among Iranian children and adolescents and other findings as well regarding Tehranian populations^24,25^. Compared to previous reports, the longitudinal structure of the present study, which provides an opportunity to follow participants and track their BMI trend over a long duration after childhood would add to the value of the current study; this finding might be explained by different processes of growth and developmental in boys and girls, and emphasizes some psychological and socio-cultural conditions leading to different weight expectations and body images among teenage boys and girls. In this regard evidence shows that while teenage girls tend to have lower body weight and look beautiful, boys tend to increase their muscle and body weight and portray stronger body images^25^.

Based on the current results, high-risk parental cluster was associated with higher BMI in children at all percentiles for all ages during the follow up duration, findings consistent with those of another study that showed the positive effect of obesogenic familial environments on the BMI levels of female children^20,21^. Furthermore, several studies have been conducted to investigate individual effects of parental factors on the entire distribution of BMI using quantile regression analysis. Two of these emphasized the effect of maternal employment on the upper percentiles of BMI among Chinese children and adolescents^26,27^, findings which could confirm our results regarding the contributing role of mothers’ employment to define the high-risk parental cluster and predisposing children to higher BMI. McDonnell and Doyle report that employment only among well-educated mothers increases the risk of overweight and obesity in pre-school Irish children^28^; their findings showed that maternal employment (both part- and full-time) had greater effect on the upper percentiles of the children’s weight only in highly educated mothers, findings consistent with those of two German studies demonstrating the effect of parental education, especially mothers, on the BMI distribution of their children^29,30^.

Interestingly, in the current study, levels of education in mothers and fathers revealed a paradoxical prognostic effect on their children’s BMI percentiles. While none of the mothers in the low-risk parental cluster were highly educated, fathers with high education were mostly placed in the low-risk parental cluster, implying different social roles and expectations for women and men in different societies, e.g. while increased levels of fathers’ education can improve the socioeconomic status of the family and lifestyles of the children, higher education levels of mothers’ education predispose children to the increasing risk of obesity.

Considering the sex-specific effect of mothers’ and fathers’ education levels, Kim *et al*. demonstrated the association of parental education with percentiles of BMI distribution among Korean adolescents^31^; their study showed that higher levels of education in fathers and mothers was associated with lower BMI for girls and higher BMI for boys respectively. Although educated mothers have higher levels of knowledge on the nutritional and health needs of their children, most of them are employed; combining employment and the parenting role makes it difficult to maintain the attention and care given to children and has a major impact on the family lifestyle. Employed mothers have to spend more time outside the home and hence have limited time to supervise their kids, prepare healthy foods and encourage/accompany their children to physical activity sessions. These children, more often than not, end up spending more time watching TV, less on physical activity, and may have to suffer negative consequences health wise^32^. This issue would be more prominent in Iran, which is undergoing a transition from traditional culture to modernization, demonstrated by the steep escalating trend in urbanization^33^.

Regarding the association of maternal obesity with childhood BMI over the entire distribution, there is much evidence emphasizing the impact of maternal BMI on the upper percentile of BMI distribution among Chinese and German children^16,27,29,30,34^, which underscores the current findings regarding the contributing role of maternal obesity in defining high-risk parental cluster and predisposing children to higher levels of BMI.

This study has both strengths and limitations. As a family-based cohort study conducted on a West-Asian community, the current study provides a unique opportunity for long-term tracking of BMI status among a large population of school-aged children based on their parental characteristics. To achieve this goal, in this study, for the first time, the synergistic effect (not individual) of parental characteristics on entire distribution (different percentiles) of childhood BMI has been investigated. However, the current study has focused on urban families and the results may not be generalized to rural areas. In addition, to assess and explore parental risk clusters, data on some potentially effective variables, including parental psychological characteristics and family income were not available and these need to be considered in the future research.

## Conclusion

Findings of the present study indicate the synergistic effects of parental factors on different percentiles of BMI distribution. Our data indicated that during follow up and at all ages, children of high-risk families were more likely to have higher BMIs than those of low-risk families, particularly in the upper percentiles of BMI distribution. By identifying the most vulnerable families and children, our results can provide valuable information for designing and implementing more cost-effective community-based strategies and programs to monitor and control weight in the early years of life.

## Methods

### Study design

This study was conducted within the framework of the Tehran Lipid and Glucose Study (TLGS), an ongoing community-based prospective study of a representative sample of residents of district 13 in Tehran, Iran. The TLGS is divided into two phases: A cross-sectional study of the prevalence of non-communicable diseases such as diabetes and cardiovascular disease and their associated risk factors, and a prospective follow-up study. From a total of 20 medical health centers located in district 13, three centers were randomly selected, from which initially a total of 27,000 individuals were invited to participate in the TLGS; of these invited individuals, a total of 15,005 (aged ≥3 years) agreed to participate and signed consent forms (response rate, 55.5%). The cross-sectional phase began in 1997 and was completed in 2000; the first follow-up survey began in 2001, was completed in 2005 and was then repeated every three years. Further details of the TLGS protocol and data collection process have been published previously^35,36^. This study was approved by the research ethics committee of the Research Institute for Endocrine Sciences of the Shahid Beheshti University of Medical Sciences, and all participants and/or their legal guardians provided written informed consent prior to initiation of the study. The authors confirme that all research was performed in accordance with relevant guidelines/regulations.

### Study population

Our sample included 2118 families who had children, aged 8–18 years, at the baseline assessment of the TLGS. A total of 2296 children, who had complete parental information at baseline were recruited for the present study and followed for the next four examinations (almost fifteen years); the quantile regression analysis sample included 1130 boys and 1154 girls, who provided complete BMI data across all follow-up visits.

### Outcome assessment

The outcome of this study was the BMI status (Kg/m^2^) of children which was considered a continuous variable in the current analysis process. BMI was calculated as the weight (Kg) divided by the height (m) squared.

### Clinical and laboratory measurements

Based on the TLGS measurement protocol^35,36^ weight was measured while wearing minimum clothing and no shoes, using a digital scale; height was measured without shoes, in a standing position with shoulders in normal alignment; waist circumference (WC) was measured at the umbilical level without any pressure to the body surface and was recorded to the nearest 1 cm.

After a 15-min rest in the sitting position, two measurements of systolic and diastolic blood pressure (SBP and DBP) were taken on the right arm, using a standardized mercury sphygmomanometer (calibrated by the Iranian Institute of Standards and Industrial Researches); the mean of the two measurements was considered as the participant’s blood pressure.

A blood sample was taken between 7:00 and 9:00 AM from all study participants, after 12 to 14 h of overnight fasting. All blood analyses were carried out at the TLGS research laboratory on the day of the blood collection. Details of laboratory measurements including levels of fasting blood glucose (FBG), triglycerides (TG), high-density lipoprotein cholesterol (HDL-C) have been reported previously^35,36^.

### Definition of terms

Baseline parental characteristics including age, education, employment status, physical activity and smoking, general obesity and metabolic syndrome of both mothers and fathers were considered in the present study. Education was categorized at four levels: (1) illiterate, (2) primary, (3) secondary and (4) higher. Employment status has been defined as (1) employed, (2) unemployed and (3) unemployed but having income. Physical activity was categorized as (1) low-, (2) moderate- and (3) high activity. Smoking habits were considered in three groups: 1) non-, 2) occasional- and 3) daily smokers. General obesity was determined as (1) normal weight (BMI < 25 kg/m^2^), (2) overweight (BMI ≥ 25 kg/m^2^ and <30 kg/m^2^) and obesity (BMI ≥ 30 kg/m^2^). Metabolic syndrome (MetS) was determined using the joint interim statement, which defines MetS as the presence of any three of the following five risk factors: (1) Abdominal obesity with WC ≥ 90 cm for both sexes^37^; (2) reduced HDL-C < 50 mg/dl in women, <40 mg/dl in men, or receiving drug treatment for reduced HDL-C; (3) elevated TG levels ≥ 150 mg/dl or receiving drug treatment for elevated TG; (4) elevated blood pressure (≥130 mmHg SBP or ≥85 mmHg DBP) or a patient with a history of hypertension, receiving antihypertensive drug treatment and (5) elevated FBG ≥ 100 mg/dl or receiving drug treatment for elevated FBG levels^38^.

### Statistical analysis

To achieve the first goal of the current study, two-step cluster analysis was conducted to identify parental clusters based on the potential weight-related characteristics of both mothers and fathers. Two step cluster analysis, as a model based method for the classification of subjects was used for both continuous and categorical variables and inherent differences between subjects were detected^39^. In order to verify the internal consistency of the clustering procedure the explored clusters were compared based on the parental characteristics, initially classified. One way ANOVA and chi-square test were used to compare the continuous and categorical parental variables between clusters, respectively. To determine distribution of the children’s BMI in each cluster, age- and sex-specific curves of specific percentiles (5%, 50%, 85%, 95% and 97%) were applied using the Lambda-Mu-Sigma (LMS) method^40,41^ in the Vector Generalized Linear and Additive Models (VGAM) package R version 2.15.1 (R Development core team, Vienna Austria). Due to the longitudinal structure of the present study (repeated observations of BMI taken on the same individual over time), individual effects included in the underlying models and Linear Quantile Mixed Models (LQMM) were used to compare BMI distribution among clusters at the 5^th^, 25^th^, 50^th^, 75^th^, 85^th^, 95^th^ and 97^th^ percentiles of children’s BMI^42^. For these aims, two sex-specific models were run as follows. Model 1 was only adjusted for children’s age (centered at 13 years for both sexes); model 2 was adjusted for cluster status as well as children’s age. LQMM package R version 2.15.1 (R Development Core Team, Vienna, Austria) was used to estimate the aforementioned models.

## Data Availability

Due to confidentiality conditions, the authors were only allowed to publish analytic results from the data, but not the data itself. Data may be available on request to the last author.
